# Extrasynaptic GABA_A_ receptors in mediodorsal thalamic nucleus modulate fear extinction learning

**DOI:** 10.1186/1756-6606-7-39

**Published:** 2014-05-29

**Authors:** Afshin Paydar, Boyoung Lee, Gireesh Gangadharan, Sukchan Lee, Eun Mi Hwang, Hee-Sup Shin

**Affiliations:** 1 Center for Cognition and Sociality, Institute for Basic Science (IBS), 70, Yusung-daero 1689-gil, Yusung-gu, Daejeon 305-811, Republic of Korea; 2 Department of Neuroscience, University of Science and Technology (UST), 217 Gajeong-ro Yuseong-gu, Daejeon 305-333, Republic of Korea; 3 WCI Center for Functional Connectomics, Korea Institute of Science and Technology, Seoul 136-791, Republic of Korea

**Keywords:** Fear extinction, Mediodorsal thalamus, Extrasynaptic GABA_A_ receptor, GABRA4, Tonic GABA inhibition, Anxiety disorders

## Abstract

**Background:**

The gamma-amino-butyric acid (GABA) system is a critical mediator of fear extinction process. GABA can induce “phasic” or “tonic” inhibition in neurons through synaptic or extrasynaptic GABA_A_ receptors, respectively. However, role of the thalamic “tonic GABA inhibition” in cognition has not been explored. We addressed this issue in extinction of conditioned fear in mice.

**Results:**

Here, we show that GABA_A_ receptors in the mediodorsal thalamic nucleus (MD) modulate fear extinction. Microinjection of gabazine, a GABA_A_ receptor antagonist, into the MD decreased freezing behavior in response to the conditioned stimulus and thus facilitated fear extinction. Interestingly, microinjection of THIP (4,5,6,7-tetrahydroisoxazolo[5,4-c]pyridin-3-ol), a preferential agonist for the δ-subunit of extrasynaptic GABA_A_ receptors, into the MD attenuated fear extinction. In the opposite direction, an MD-specific knock-out of the extrasynaptic GABA_A_ receptors facilitated fear extinction.

**Conclusions:**

Our results suggest that “tonic GABA inhibition” mediated by extrasynaptic GABA_A_ receptors in MD neurons, suppresses fear extinction learning. These results raise a possibility that pharmacological control of tonic mode of GABA_A_ receptor activation may be a target for treatment of anxiety disorders like post-traumatic stress disorder.

## Background

A failure in inhibition of maladaptive associative responses to environmental stimuli is a hallmark of anxiety disorders such as phobias and post-traumatic stress disorder (PTSD). Dysfunctional neural responses underlie such disorders [1]. Fear extinction in animals is used for understanding these disorders and developing methods for their treatment. In fear extinction repetitive exposures of a fear-conditioned animal to the conditional stimulus (CS) in the absence of the unconditional stimulus (US) will result in a decrease of the conditional response (CR), freezing behavior [1-3]. An impairment of fear extinction has been reported in several mouse mutants [4,5], providing an opportunity where the neural mechanism underlying PTSD may be delineated.

GABA has an essential role in fear extinction through modulating cellular activities in the amygdala, medial prefrontal cortex (mPFC), and hippocampus, key structures involved in fear extinction [3,6-12]. However, there has been controversies regarding the exact role of GABAergic cells or receptors in fear extinction [2,6]. For a better understanding of detailed mechanisms of fear extinction, the role of the GABAergic system, especially in its control of neuronal firing modes, needs to be elucidated.

The inhibition in thalamocortical (TC) neurons is mediated predominantly through ionotropic GABA_A_ receptors (GABA_A_Rs) [13,14]. TC neurons exhibit two different modes of GABA_A_R-mediated inhibition; a transient “phasic inhibition” through α1β2γ2 synaptic receptors, and a continuous “tonic inhibition” mediated by α4β2δ extrasynaptic GABA_A_R (eGABA_A_Rs) [14-16]. Modulation of GABA_A_Rs activities, especially eGABA_A_Rs, contributes to the shift between different firing modes in the thalamus, and promotes the transition between different behavioral states in absence seizure animal models [17-20]. However, the physiological role of thalamic tonic GABA inhibition in cognitive behaviors is not known.

The mediodorsal nucleus (MD) of thalamus, a part of the TC system in the thalamus, receives GABAergic afferents from multiple brain regions including ventral pallidum, substantia innominata, globus pallidus, nucleus reticularis thalami and substantia nigra pars reticulata [21,22]. The MD has interconnections with the key regions of the fear extinction circuits, mPFC and amygdala [21-23]. Unilateral bicuculline infusion into the MD induces ipsi- and contralateral c-fos expression in the mPFC [24]. MD neurons exhibit dual modes of firing, burst or tonic, and the firing mode of MD neurons can modulate fear extinction bidirectionally; tonic firing facilitates, whereas burst firing attenuates extinction [5]. Tonic firing results from membrane depolarization, while burst firing is induced by membrane hyperpolarization following inhibitory inputs. However, the details of the mechanisms for inducing hyperpolarization of MD neurons in fear extinction suppression have not been defined.

In this study, we investigated the role of eGABA_A_Rs in the MD in fear extinction using pharmacological and genetic tools in mice. We found that the extrasynaptic GABA_A_Rs in the MD suppress fear extinction learning.

## Results

### Injection of gabazine into the MD facilitates fear extinction

**Figure 1:**
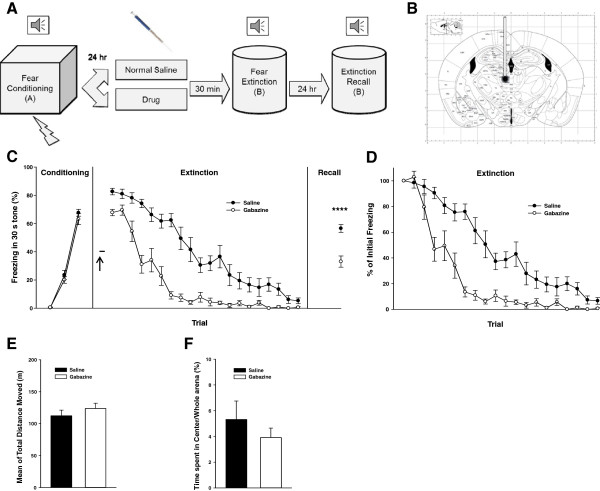
**Gabazine injection into the MD facilitates fear extinction. (A)** experiment scheme. **(B)** Injection target. **(C)** Injection of gabazine (30 μM) into the MD (empty circles, *n* = 12) 30 min before extinction decreased freezing levels in both extinction learning, and the recall test in the next day compared with the mice that received vehicle injection into the MD (filled circles, *n* = 11), ↑, injection time point. **(D)** Normalized within-session extinction to percentage of the initial freezing levels of each group shows group difference and group x trial interaction in extinction rate. **(E and F)** No difference between these two groups in the locomotion, or anxiety. Data are means ± SEM.

To test the effect of GABA_A_Rs activities in the MD on fear extinction learning, we injected gabazine, a GABA_A_R inhibitor, into the MD of wild-type mice (Figure 1A-B). In day 1 (conditioning), we exposed the cannula-implanted mice to three trials of tone (CS), each co-terminated with an electric foot shock (US). In day 2 (extinction learning), 30 min after injection of gabazine or vehicle through the cannula, the fear-conditioned mice were exposed to 20 repeated CS-only trials. In the extinction learning gabazine-injected mice showed a faster decrease in freezing levels compared to the mice in the vehicle-injected group (two-way repeated measures ANOVA, group effect, F1,21 = 71.745, P < 0.001; group × trial interaction, F21,399 = 5.414, P < 0.001; Figure 1C). In comparison with the mice in the vehicle-injected group, the gabazine-injected mice showed lower initial levels of freezing (*t* test; P = 0.0001; Figure 1C). Therefore, to compare the rates of within-session extinction between the two groups, the freezing levels for trials in each group were normalized to the value for the first trial of each group (Figure 1D) following the convention [25,26]. There was a difference between groups (two-way repeated measures ANOVA, group effect, F1,21 = 55.024, P < 0.001; group × trial interaction, F21,399 = 5.318, P < 0.001), indicating that gabazine injection into the MD has changed the extinction rate. In the third day (extinction recall), mice were re-exposed to the CS without the US. The gabazine-treated group showed significantly lower freezing levels than the vehicle-injected group (*t* test, P = 0.0001; Figure 1C), indicating that gabazine facilitated extinction learning in the mice.

To rule out the possible confounding effect of GABA_A_Rs activity in the MD on the locomotion and anxiety level of the mice, we injected gabazine or vehicle into the MD of the mice 30 min before performing an open field test. The two groups did not differ in total distance moved during a 1 hr period in the open field arena (*t* test, P = 0.365; Figure 1E), and showed no significant difference in the percentage of time spent in the central area compared to the whole arena in the first 5 min (Mann–Whitney Rank Sum Test, P = 0.758; Figure 1F), indicating that the lower freezing response of gabazine-injected mice was not the result of an increased locomotor activity in the extinction box or decreased anxiety.

### Injection of THIP into the MD attenuates fear extinction

**Figure 2:**
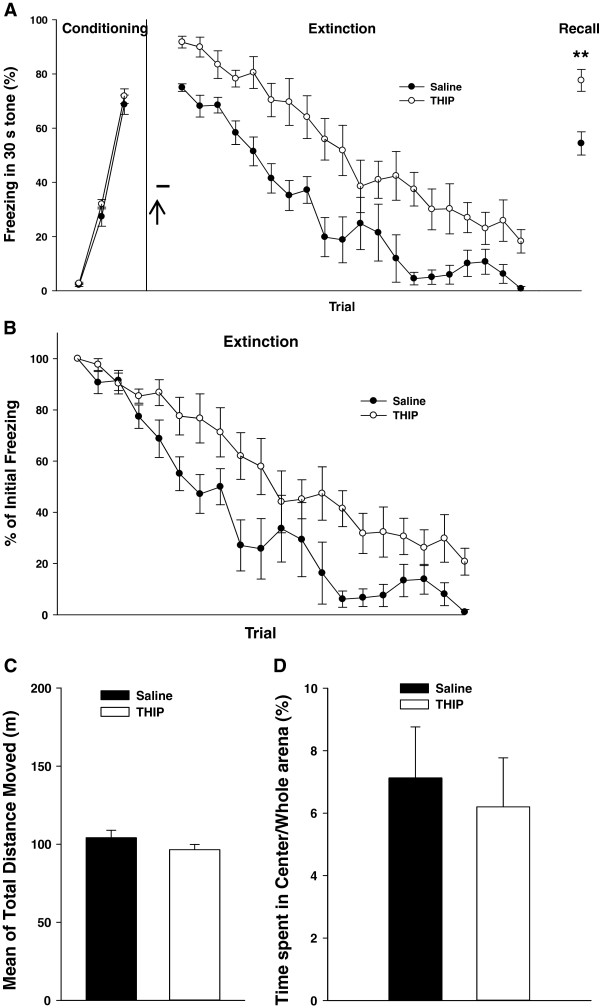
**THIP injection into the MD attenuates fear extinction. (A)** THIP injection (50 μM) into the MD (empty circles, *n* = 11) 30 min before extinction increased freezing levels in both extinction learning, and a day after that in the recall test compared with the mice that received vehicle injection into the MD (filled circles, *n* = 7), ↑, injection time point. **(B)** Normalizing within-session extinction to percentage of initial freezing levels of each group shows a difference in extinction rate between the two groups. **(C and D)** There was no difference between the two groups in the locomotion or anxiety level. Data are means ± SEM.

The α4β2δ eGABA_A_Rs are highly expressed in the thalamus including the MD [27,28]. To test whether eGABA_A_Rs activity in the MD could affect fear extinction learning, we injected THIP (4,5,6,7-tetrahydroisoxazolo[5,4-c]pyridin-3-ol), a selective GABA_A_R agonist with a preference for δ-subunit containing eGABA_A_Rs [29], into the MD 30 min before fear extinction learning. During the extinction learning, the THIP-injected mice showed less decrease in freezing levels than the vehicle-injected group (two-way repeated measures ANOVA, group effect, F1,16 = 32.315, P < 0.001; group × trial interaction, F16,304 = 0.665, P < 0.852; Figure 2A). Due to higher freezing levels in the first trial in the THIP-injected group compared with the vehicle-injected group (*t* test; P = 0.00003; Figure 2A), we further examined the rates of within-session extinction between the two groups by normalizing freezing levels to that of the first trial for each group (Figure 2B) following the convention [25,26]. There was significant difference between the two groups in the rates of within-session extinction (two-way repeated measures ANOVA, group effect, F1,16 = 12.127, P = 0.003; group × trial interaction, F16,304 = 1.065, P < 0.387). This indicates that THIP injection changed within-session extinction rate. In the extinction recall, the THIP-treated group showed significantly higher freezing levels than the vehicle-injected group (*t* test, P = 0.002; Figure 2A), indicating that THIP attenuated extinction learning in the mice.

To check the possible effect of eGABA_A_Rs activity in the MD on the locomotion and anxiety level of the mice, we injected THIP or vehicle into the MD of the mice 30 min before performing an open field test. The two groups did not differ in locomotion during a 1 hr period (*t* test; P = 0.200; Figure 2C), and showed no significant difference in the percentage of the time spent in the central area compared to the whole arena in the first 5 min (*t* test; P = 0.702; Figure 2D), which means that the higher freezing response of the THIP-injected mice was not due to a change in the locomotor activity or anxiety.

### Conditional Knock-out (KO) of *GABRA4* in the MD facilitates fear extinction

**Figure 3:**
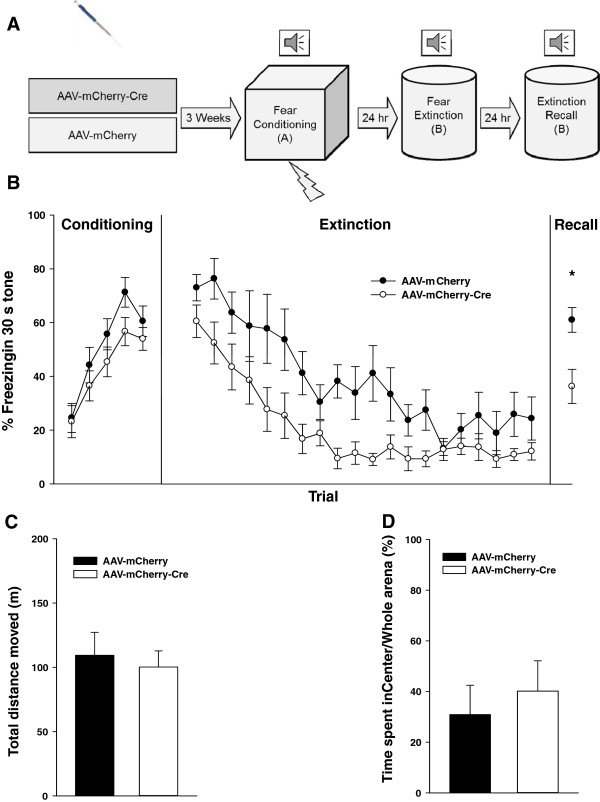
**Virus-mediated conditional KO of *GABRA4 *in the MD facilitates extinction. (A)** Experiment scheme. **(B)** The GABRA4^*flx/flx*^ mice injected with AAV-mCherry (filled circles, *n* = 6) or AAV-mCherry-Cre (empty circles, *n* = 11) into the MD showed no difference in fear conditioning. The Cre-virus injected group showed significantly lower freezing levels in the extinction learning and the recall test in comparison to the control virus injected group. **(C and D)** The two groups did not differ in the locomotion or anxiety. Data are means ± SEM.

**Figure 4:**
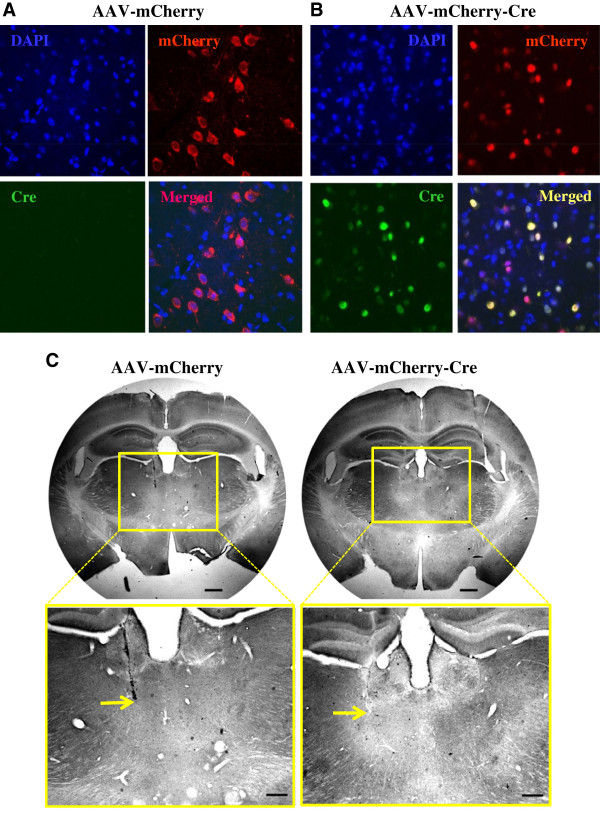
**Cre expression and *GABRA*4 KO in the transfected MD cells. (A and B)** Opposite to the MD cells from the mice injected with AAV-mCherry, immunofluorescence revealed Cre expression (green) in the mCherry-expressing cells in the MD region of the mice injected with AAV-mCherry-Cre. **(C)** A decrease of GABRA4 expression can be seen in the injected side of the MD in the Cre virus-injected mice compared with the contralateral non-injected side or the control virus-injected mice. Arrows indicate the injection sites. Scale bars: 500 μm (top), and 250 μm (bottom).

As thalamic expression of the α4-subunit is limited to the extrasynaptic area [16,27], we tested the behavioral effect of MD-specific KO of *GABRA4* gene in the fear extinction paradigm. AAV-mCherry (control virus) or AAV-mCherry-Cre viral particles were injected into the MD of GABRA4^*flx/flx*^ mice 3 weeks before fear conditioning (Figure 3A). Immunohistochemistry was used to test Cre expression and functionality. Cre expression can be seen in the mCherry-expressing cells in the MD region of the mice injected with AAV-mCherry-Cre (Figure 4A-B). A lack of GABRA4 expression in the injected side of the MD in brain slices from the Cre virus-injected mice is evident when compared with the contralateral non-injected side or slices from the control virus-injected mice (Figure 4C).

There was no difference between the two groups in fear conditioning (two-way repeated measures ANOVA, group effect, F1,15 = 4.109, P = 0.061; group × trial interaction, F15,60 = 0.365, P = 0.832; Figure 3B). There was no significant difference in the initial freezing levels between the two groups (*t* test; P = 0.186; Figure 3B). However, the mice injected with AAV-mCherry-Cre showed faster extinction learning compared to the control group (two-way repeated measures ANOVA, group effect, F1,15 = 9.947, P = 0.007; group × trial interaction, F15,285 = 1.111, P = 0.338; Figure 3B). In the extinction recall test, the group injected with AAV-mCherry-Cre showed significantly lower freezing levels than the control virus-injected group (*t* test; P = 0.018; Figure 3B), indicating that *GABRA4*-deletion facilitated extinction learning in the mice.

The two groups did not differ in locomotion (*t* test, P = 0.679; Figure 3C) or the percentage of the time spent in the central area compared to the whole arena in the first 5 min (Mann–Whitney Rank Sum Test, P = 0.880; Figure 3D), indicating that the lower freezing response of the Cre virus-injected mice was not the result of increased locomotor activity or decreased anxiety.

## Discussion

In this study we found that activity of GABA_A_Rs, especially α4- and δ-containing eGABA_A_Rs in the MD can suppress fear extinction. As we previously reported the firing mode of MD neurons modulates fear extinction bidirectionally; tonic firing facilitates, while burst firing attenuates extinction learning [5]. Individual action potentials of tonic firing occurs during membrane depolarization, while burst firing is induced by low-threshold calcium spikes mediated by low-voltage activated T-type calcium channels that are activated by membrane hyperpolarization following inhibitory inputs [13,17]. Rich GABAergic inputs to the MD can hyperpolarize neuronal membranes through the activation of α1β2γ2 synaptic or α4β2δ extrasynaptic GABA_A_Rs, resulting in a discrete “phasic inhibition” or a continuous “tonic inhibition”, respectively. This can modulate MD neurons firing pattern toward the bursting mode [14,17], and as we found attenuated fear extinction.

The GABAergic system in the amygdala [7,8], hippocampus [7,11] and mPFC [7,10] is involved in fear extinction, although there are some controversies regarding the exact roles of GABAergic cells or receptors in fear extinction [2,6]. Our results which showed the suppressive role of tonic inhibition in the MD in fear extinction may provide better insight into the detailed mechanisms underlying the GABAergic control of fear extinction.

Moreover, current findings confirm our previous study showing the role of the MD in fear extinction [5]. Consistently, elevated metabolism in the MD has been observed in relation to fear extinction [30]. Furthermore, it has been shown that prefrontal long-term potentiation induced by high-frequency stimulation of the MD facilitates retention of extinction, while prefrontal long-term depression induced by low-frequency stimulation of the MD has the opposite effect [31].

In our pharmacologic interventions (microinjection of gabazine or THIP into the MD) we found significant differences in the initial freezing levels in extinction between the drug-injected and vehicle-injected groups. This could show a potential role of the MD in fear memory retrieval and/or the fear response itself. Therefore, to avoid this confounding effect and evaluate the extinction more precisely, we compared the rates of within-session extinction between the two groups by normalizing the freezing levels for trials in each group to the value for the first trial of that group following the convention [25,26]. There were significant differences between the two groups in the rates of within-session extinction (Figures 1D and 2B). These results indicate that gabazine or THIP injection changed rate of within-session extinction, confirming the role of the MD in fear extinction. However, in the case of *GABRA4* conditional KO there was no significant difference in the initial freezing levels between the two groups. The different effects on the first trials of extinction between pharmacologic and genetic interventions could be due to the fact that drug effects are acute, while the gene KO is long lasting and can induce compensatory changes. Moreover, drugs usually have effects on other receptor subtypes and are not completely specific, while *GABRA4* KO is specific to eGABA_A_Rs in the MD. Nevertheless, these results show that there are indeed differences in extinction between the interventions and control groups. Furthermore, we clearly showed differences between the groups in extinction retrieval experiments, which test the extinction memory. Taken together, these show that the extrasynaptic GABA_A_Rs in the MD modulate fear extinction.

We found that inhibition of the GABA_A_Rs in the MD through microinjection of gabazine facilitated fear extinction (Figure 1C). According to Cope et al. (2005) gabazine promotes tonic firing in the TC cells. Therefore, increase of tonic firing by inhibition of GABA_A_Rs in the MD, a subregion of TC nuclei, would facilitate acquisition of fear extinction [17]. Using electrolytic lesions, Garcia et al. (2006) did not find any role of the mPFC and MD in fear extinction [32]. Interestingly, the same group has a contrary report, showing that MD-mPFC transmission and plasticity has a role in fear extinction [33]. Moreover, shortcoming of electrolytic lesion is that it destroys all cell types and passing fibers permanently, thus producing a compound effect [34].

On the other hand, we showed that the activation of eGABA_A_Rs in the MD using THIP attenuates fear extinction (Figure 2A). It is well known that TC neurons show a persistent “tonic inhibition” through α4β2δ eGABA_A_Rs [14,35]. As reported previously, THIP, a δ-subunit preferring GABA_A_R agonist, enhances tonic inhibitory currents in TC neurons and promotes burst firing by hyperpolarizing TC neurons [17]. Therefore, increase of burst firing through the activation of eGABA_A_Rs would suppress acquisition of fear extinction; thus increasing the tonic inhibition attenuates fear extinction. These results are consistent with our previous work on the bidirectional modulation of fear extinction by thalamic firing modes [5]. However, Padilla-Coreano et al. (2012) reported no change in fear extinction memory with the injection of muscimol, a GABA_A_R agonist, into the midline thalamus, including the MD, paraventricular, and centromedial nuclei, in rats [36]. It has been reported that muscimol can show opposite effects at different concentrations [37]. Therefore, this difference could also be due to injection of a high concentration of this drug in the study mentioned above.

Lack of tonic inhibition in the TC neurons in GABA_A_R α4-subunit (*GABRA4*) KO mice and exclusively extrasynaptic location of α4-subunits in this region [27] makes α4-subunit containing eGABA_A_Rs an excellent tool for studying the effect of the removal of tonic inhibition in the thalamus. We found that virus-mediated conditional KO of *GABRA4* in the MD facilitates extinction learning. Interestingly, this finding is also in concordance with our previous report showing the role of the MD in fear extinction [5].

## Conclusions

Our results obtained by pharmacological and genetic tools show the involvement of GABAergic system in the MD in fear extinction, especially through the eGABA_A_Rs. Our findings suggest that “tonic GABA inhibition” mediated by α4β2δ eGABA_A_Rs in MD neurons, suppresses fear extinction learning. This provides further support to the idea of the thalamic modulation of fear extinction [5] and identifies the eGABA_A_Rs as a key mediator in this process.

Anxiety disorders, such as PTSD, panic disorder and phobias have a substantial psychiatric and social burden for both the patients and society, which calls for extensive clinical interventions for treatments [12]. Research advancements in neural mechanisms of fear extinction are promising to provide a way for improving these therapeutic methods. As the GABAergic system is known to be a key player in fear extinction, these novel observations regarding the modulatory effect of eGABA_A_Rs in the MD on fear extinction may propose a potential target for developing new therapeutic methods, or provide a potential mechanism for existing treatments for conditions like PTSD.

## Methods

### Mice

Adult (12–16 weeks old) male wild-type F1 (B6 × 129) hybrid mice, used for the drug infusion experiments, were obtained by mating parental strains of C57BL/6J and 129s4/svJ. For the virus injection experiments, adult male (12–16 weeks old) B6.GABRA4^*flx/flx*^ mice were used. B6.GABRA4^*flx/flx*^ mice were produced by mating B6.GABRA4^*flx/+*^mice. Mice had *ad libitum* access to food and water and were housed under a 12:12 hr light/dark cycle (lights on at 8 AM). Animal care and experiments followed the guidelines from the Institutional Animal Care and Use Committee of Institute for Basic Science.

### Fear conditioning and extinction

The mice went through fear conditioning, extinction, and extinction recall as described previously [5]. Briefly, the mice were conditioned in conditioning chamber (context A) using three trials of CS + US: tones (3 KHz, 30 s, and 85 dB) co-terminating with foot shocks (0.35 mA, 1 s). For the B6GABRA4^*flx/flx*^ mice, five trials of tone and foot shock (0.7 mA) were used. The intertrial interval was 120 s. After 24 hr, mice were exposed to twenty CS-only (tone) trials in the extinction box (context B) with an intertrial interval of 5 s. For the extinction recall test, mice were exposed to the tone on the third day (context B).

Mice behavior was recorded with a video camera to score freezing (lack of movement except for respiration) using the FreezeFrame software (Actimetrics, Coulbourn Instruments, PA, USA).

### Open field test

Locomotor activity was tested in an open field box (40×40×50 cm) as reported previously, one week apart from fear conditioning and extinction [5]. The ratio of the time spent in the central 20×20 cm area to the time spent in the whole 40×40 cm arena in the first 5 min was used as a measure of anxiety. Automated measurements were done using EthoVision software (Noldus Information Technology, Netherlands).

### Drug delivery

Mice were anesthetized using intraperitoneal injection of 2% Avertin solution (tribromoethyl alcohol/tertiary amyl alcohol, vol/vol, Sigma-Aldrich), and framed in stereotaxic device (Kopf instruments, CA, USA). Mouse surgery for unilateral implantation of guide cannula was done according to the protocols [5], targeting the MD (AP: −1.5 mm, ML: 0.3 mm, and DV: −3.3 mm from the brain surface). Gabazine or THIP (from Sigma-Aldrich, St. Louis, MO, USA) were dissolved in normal saline, and a volume of 0.3 μl (30 and 50 μM, respectively) was infused (0.1 μlmin^−1^) through a 33-gauge injection cannula inserted in the guide cannula. The injector was kept in place for 3 min after the end of the injection. Histologic confirmation of the infusion position was carried out and only the data from properly injected mice were used for statistical analysis.

### Production and injection of recombinant AAV-mCherry-Cre and AAV-mCherry

For AAV2 production, we used the pAAV-MCS plasmid (Stratagene, La Jolla, CA) carrying the cDNA for mCherry-Cre (NLS-Cre was kindly provided by Dr. C. Justin Lee, KIST) or mCherry downstream of the CMV promoter. For recombinant virus generation, AAV-293 cells were co-transfected with pAAV-RC (Stratagene) encoding the AAV genes rep and cap and the helper plasmid (Stratagene) encoding E24, E4 and VA. Virus particles were purified and concentrated according to the procedure [38]. The AAV viral titer was determined using a QuickTiterTM AAV Quantitation Kit (Cell Biolabs, San Diego, CA) and a minimum titer of 10^11^/ml was obtained. The AAV-mCherry control or AAV-mCherry-Cre virus was injected in the MD region (AP: −1.5 mm, ML: 0.3 mm, and DV: −3.3 mm from the brain surface) of B6GABRA4^*flx/flx*^ mice unilaterally, using a Hamilton syringe connected to a microinjection pump (sp100i instrument, WPI, USA) with a volume of 0.6 μl at a rate of 0.1 μlmin^−1^. The needle was held in place for 10 min after completion of the injection to permit the diffusion of the viral particles into the brain tissue.

### Immunohistochemistry and immunofluorescence

To check Cre and α4-subunit expression in the MD, we performed histology as described previously [39]. Mice were perfused transcardially with cold saline, followed by 4% paraformaldehyde in phosphate-buffered saline (PBS) (10 mM, pH 7.4) under Avertin anesthesia. Brains were postfixed in 4% paraformaldehyde overnight at 4°C. Coronal sections (50 μm thickness) of the MD were prepared using a vibratome (VT1200S, Leica, Germany).

For diaminobenzidine (DAB) immunostaining, free-floating sections were initially washed (3×) in PBS, and then cell membranes were permeabilized using PBS supplemented with 0.1% Triton X-100 (PBST) for 10 min and then incubated in 0.3% H_2_O_2_ in PBS (20 min). Next, tissue was blocked (1 h) in 5% goat serum⁄PBS and incubated (overnight, 4°C) in a rabbit polyclonal anti α4-subunit antibody (1:1000 final dilution, ab4120 abcam, UK). After 3× washes in PBS, sections were incubated (2 h) at room temperature (25°C) in biotinylated anti-rabbit IgG (1:500, Vector Laboratories, Burlingame, CA, USA) and then placed in an avidin⁄biotin horseradish peroxidase complex for 1 h (prepared according to the manufacturer’s instructions, Vector Laboratories). The signal was visualized by the addition of diaminobenzidine-nickel-intensified substrate (Vector Laboratories, USA), mounted on gelatin-coated slides and coverslipped with Permount media (Fisher Scientific, Houston, TX, USA).

For fluorescent immunolabeling against Cre, tissue sections were incubated (overnight, 4°C) with mouse monoclonal anti-Cre antibody (1:1000, MAB3120, EMD Millipore, Germany). The sections were washed with PBS and then incubated (2 h at room temperature) with an Alexa 488-conjugated anti-mouse IgG antibody (1:500, Molecular Probes, Eugene, OR, USA). Sections were washed in PBS, and then incubated (30 min) with the DNA stain 4′,6-diamidino-2-phenylindole (DAPI) (Thermo Scientific, USA), washed in PBS again, and then mounted with VECTASHIELD (Vector Laboratories, Inc. Burlinggame, CA, USA).

Images of diaminobenzidine-labeled sections were captured using a light microscope (ECLIPSE T*i*, Nikon, Japan). For fluorescent imaging, a confocal microscope (IX81, Olympus, Japan) was used.

Figures were prepared using Adobe Photoshop CS6 (Adobe Systems Inc., USA) and Microsoft Office 2010 (Microsoft Corporation, Redmond, USA). Image manipulation was restricted to threshold and brightness adjustments applied to the entire image.

### Data analysis

Making graphs, two-way repeated measures ANOVA, *t* test or equivalent non-parametric tests were done using the Sigmaplot software (Systat Software Inc., USA).

## Competing interests

The authors declare that they have no competing interests.

## Authors’ contributions

AP, SL, and H-SS designed the experiments; AP, BL, GG, and EMH, performed the experiments; AP and SL analyzed the data; AP and H-SS wrote the paper. All authors read and approved the final manuscript.
